# Short-range disorder in MOF glasses

**DOI:** 10.1093/nsr/nwaa207

**Published:** 2020-09-02

**Authors:** Ming-Hua Zeng

**Affiliations:** 1 Key Laboratory for the Chemistry and Molecular Engineering of Medicinal Resources, School of Chemistry and Pharmaceutical Sciences, Guangxi Normal University, China; 2 Hubei Collaborative Innovation Center for Advanced Organic Chemical Materials, Ministry of Education Key Laboratory for the Synthesis and Application of Organic Functional Molecules, Hubei University, China

Glass appears to be an ordinary material with wide applications in daily life and industry; however, its formation mechanism, phase transition and microscopic structure remain mysterious. It is accepted to be an amorphous solid exhibiting glass transition, with short-range (<6–8 Å) order, but long-range (>20 Å) disorder structures [1]. Experimental determination of the structure of glass is crucial to understand its dynamic and physical properties. ‘What is the nature of glass?’ is one of the most fundamental and challenging scientific questions [1].

Recently, in addition to traditional inorganic, metallic and organic glasses, a fourth category of glass has been identified, derived from crystalline metal-organic frameworks (MOFs) and the subset of zeolitic imidazolate frameworks (ZIFs). Understanding this newcomer to the glass world is fundamentally important in attempts to understand the ‘bigger picture’ of the nature of glass [1–4]. Very recently, an international team led by Prof. Y. Z. Yue and Prof. S. Sen revealed the short-range structure within MOF glass, using solid-state ^67^Zn MAS NMR with the most powerful magnetic field of 35.2 T at the National High Magnetic Field Laboratory (NHMFL) [5]. They analyzed experimental NMR spectra of both crystalline and glassy samples derived from ZIF-4 and ZIF-62 (Fig. 1). Two Zn sites with different distortions were identified in the ZIF crystals. Significant broadening and lowering of chemical shifts for the ^67^Zn resonance peaks were observed in the ZIF glass, indicating great geometry distortion of the Zn[ligand]_4_ tetrahedra. Melting yielded a continuous Zn site and N-Zn-N angles distribution, and subsequent quenching ‘trapped’ the glass state. Thus, the network structure of the glass becomes highly disordered in short, intermediate and long ranges. Disappearance of the two distinct Zn sites characteristic of the ZIF crystals upon melting and vitrification indicates that scission and renewal of the Zn–N bonds upon melting causes structural reconstruction. The considerably weak coordination bonds and bulky nature of the organic linkers with steric hindrance in the ZIF glass synergistically limited the ability of the linker to return to its equilibrium position—to the structurally ordered state with lower potential energy.

**Figure 1.**
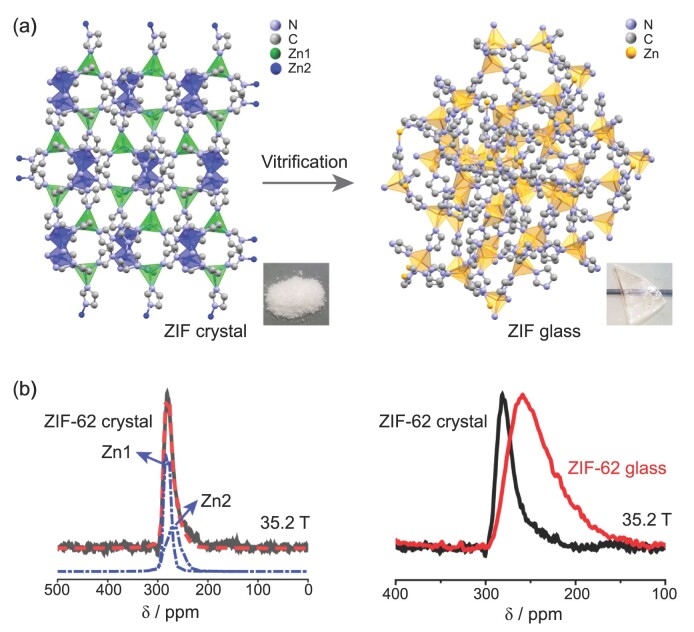
(a) Schematic representation of the structural evolution from crystalline ZIF-62 (powder) to its glassy state (transparent bulk glass) during melt-quenching [5]. (b) ^67^Zn NMR spectra of ZIF-62 crystal (left, black) and glass (right, red) collected at 35.2 T. The individual simulation spectra for Zn1 and Zn2 in ZIF-62 crystal are highlighted in dot-dashed blue lines, vertically offset for clarity.

Many known techniques have not revealed the symmetry and the degree of disorder of the Zn[ligand]_4_ tetrahedra. However, use of ultra-high magnetic field ^67^Zn NMR has overcome previous problems of probing the signals of the central ion zinc, such as low gyromagnetic ratio, large quadrupole moment and low natural abundance, as well as low atomic density of MOFs. This is no doubt a major step towards understanding the local structure of MOF glasses, and hence, the glass formation mechanism. Such a vivid example suggests that this kind of exciting research on MOF glasses has great potential to further reveal the nature of glass. Meanwhile, driven by rapid advancement in both experimental and theoretical techniques, it is anticipated that the study of MOF glass will help to solve the challenging puzzle of glass more widely.

There are >80 000 known MOFs, yet the majority thermally decompose below possible melting. An urgent mission is to establish both universal theory and general methodology to guide direct glass transition below mild temperature before MOF decomposition [1]. Doing this would provide an opportunity to pursue physical and chemical prosperity in MOF glasses comparable to those of highly developed MOF crystals. This could also provide unprecedented opportunities to explore new physical/chemical functions that are different from those known in crystalline MOFs.

## FUNDING

This work was supported by the NSFC (No. 21525101), and the BaGui Talent Program of Guangxi Province (2019AC26001).

***Conflict of interest statement*.** None declared.
